# Rate of Nondiagnostic Computerized Tomography Pulmonary Angiograms (CTPAs) Performed for the Diagnosis of Pulmonary Embolism in Pregnant and Immediately Postpartum Patients

**DOI:** 10.1155/2019/1432759

**Published:** 2019-03-26

**Authors:** Sarah Hogan, Jillian Greene, Jeffery Flemming

**Affiliations:** Memorial University of Newfoundland General Hospital, Health Sciences Centre, 300 Prince Philip Drive, St. John's NL A1B 3V6, Canada

## Abstract

**Objective:**

To evaluate the nondiagnostic rate of computed tomography pulmonary angiography (CTPA) in pregnant and postpartum patients with suspected pulmonary embolism (PE) to determine whether CTPA or ventilation-perfusion (VQ) scan should be considered first line imaging in this patient population considering their equivalent accuracy and the greater radiation exposure to proliferating breast tissue of CTPA.

**Methods:**

All pregnant/postpartum female patients between 18 and 50 years of age who had CTPA within the Eastern Health Authority between November 2012 and November 2016 were included. Each scan was evaluated for nondiagnosis based on two criteria: contrast density in the main pulmonary artery, and respiratory motion artefact. If either of these criteria were not met, the scan was labelled as nondiagnostic.

**Results:**

The nondiagnostic rate overall was 43% (*n*=83). This is similar to current literature values for rates of CTPA nondiagnosis, and comparable to the reported diagnostic quality of the reporting radiologist. This is much greater compared to rates of ventilation/perfusion nondiagnosis in comparable populations. Even in patients with normal chest radiographs, which represents the main patient group where VQ may be considered as an alternative, the nondiagnostic rate of CT is much higher.

**Conclusion:**

This is the first study to attempt to identify an objective method of determining nondiagnosis in pregnant and postpartum patients undergoing a CTPA. Our results strengthen the argument that alternative imaging should be considered when investigating for PE in this population in order to protect the proliferating breast tissue, and VQ scan should be considered especially in patients with normal chest X-rays.

## 1. Introduction

Pulmonary embolism (PE) is a rare but dangerous pathological event that can occur during pregnancy and the postpartum period [1]. The incidence of PE has been reported to be 1.3 per 10 000 pregnancies, while the case fatality rate is reported to be 3.5% [2]. PE has also been recorded as the most common direct cause of death in pregnant women, with incidence estimates ranging from 0.79 to 1.08 per 100 000 maternities over a six-year period [2]. The mortality rate of undiagnosed pulmonary embolism is estimated to be approximately 30%; however, this drops to 2–8% upon correct diagnosis [3]. Therefore, it is important to have a safe and effective diagnostic test to correctly identify the presence of pulmonary embolism in this patient population.

Computer tomography (CT) scanning is one method used to identify and diagnose pulmonary embolism. Questions have been raised, however, with respect to the dangers of exposing the active, proliferating breast tissue of young women to radiation from a CT scan [4]. Based on these concerns, it has been suggested that alternative measures such as ventilation/perfusion (VQ) scanning should be used in order to minimize radiation to maternal breast tissue [5]. A CT scan has higher radiation exposure to the mother's breast tissue compared to a VQ scan. The radiation exposure from a CT scan is estimated to be at least 2.0 rad per breast, and one mSv of radiation has been associated with an additional five cases of cancer per 100 000 patients [6]. While VQ scans have been shown to provide a higher fetal radiation dose compared to CT scan radiation [7], both are well below the threshold for fetal malformations [8] and are of less concern than the CT radiation dose to maternal breast tissue. The American College of Obstetrics and Gynecologists recommend that CT scans be used as the preferred detection method of pulmonary embolism in pregnancy due to the general “superiority” this scan, as well as lower fetal radiation dose [9]. However, a published guideline approved by the Executive and Council of the Society of Obstetricians and Gynecologists of Canada in 2014 recommends that a VQ scan be used as the preferred method of detecting pulmonary embolism in pregnant women [10].

At the Eastern Health Authority in Newfoundland and Labrador, CT and VQ are used arbitrarily at the discretion of the clinician and radiologist involved. It would be of clinical value to know how often CT scans are diagnostic for the detection of pulmonary embolism in pregnant and immediately postpartum women. If this is not a reliable test, this would lend support to the argument that alternative imaging types should be used to detect pulmonary embolism in these populations in order to protect active breast tissue from needless radiation.

## 2. Materials and Methods

This study took place in Eastern Health, at the Health Sciences Centre, St. Clare's Mercy Hospital, and the Janeway Hospital in St. John's, NL. The data was collected from radiology studies stored on the Eastern Health picture archiving and communication system (PACS).

Eastern Health is the custodian of the data, which includes Computed Tomography Pulmonary Angiography (CTPA) images.

This is a retrospective study. The records that were accessed were stored within a seven-year period between April 2010 and April 2017. A list of all female patients of child bearing age who underwent a CTPA within this timeframe was collected. 83 pregnant/postpartum patients were identified from this list of patients. We identified 89 nonpregnant/postpartum patients from the same patient list with which to compare the pregnant/postpartum population.

Once we obtained this list, we used information from Eastern Health PACS and Meditech to determine which of these patients were pregnant or within the six-week postpartum period when the scan was taken. We then assessed each scan to establish which were nondiagnostic.

We did this using the following two criteria:

1. Attenuation of the pulmonary artery <300 Hounsfield units (HU).
2. Respiratory artefact. The amount of artefact was classified as 1 for no motion, 2 for motion resulting in obscuration of the segmental vessels in any segment, and 3 for obscuration of the segmental vessels in any segments. A rank of 3 resulted in a nondiagnostic scan. Each study was reread by a Royal College of Physicians and Surgeons qualified radiologist and evaluated for the above criteria.

A measure of 300 Hounsfield units was utilized as the cutoff for contrast enhancement, as all of the fellowship trained chest radiologists in our center identified this as an appropriate cutoff, although there is no evidence for any one cutoff point. Obscuration of the segmental vessels was adopted as the nondiagnostic cutoff as recently published criteria suggests that PE in segmental vessels is not clinically significant, which has been adopted by many radiologists at our facility.

The presence of either of these criteria on a scan resulted in a categorization as non diagnostic. We also recorded whether the scan was previously flagged as nondiagnostic by the opinion of the reporting radiologist for comparison to our new categorization, as documented in the radiology report for each scan. It is important to note that the radiologists who originally documented the scans as nondiagnostic or diagnostic were blind to the objective measure of nondiagnosis. They were not aware of the pulmonary artery attenuation measurement or the respiratory artefact categorization when they interpreted the scans.

The local protocols of the two CT scanners used are as follows. For the Toshiba Aquillon One, collimation was 0.5 mm, rotation speed was 0.35 seconds, pitch was 0.813, and kVp was 120.

The effective X-ray tube current-time product was an automated tube current modulation based on a quality factor with a standard deviation of 22.5. The contrast used was Isovue 370, 60 cc. There was bolus tracking, and the slice thickness was 2 mm. For the Lightspeed VCT, collimation was 0.625 mm1T, rotation speed was 0.6 seconds, pitch was 0.9844, and kVp was 120. The effective X-ray tube current-time product was 200 mAs with automated tube current modulation. The contrast used was Isovue 370, 60 cc. There was bolus tracking, and the slice thickness was 1.25 mm.

We also recorded whether a chest X-ray was completed prior to the CTPA. The primary objective is to determine the rate of nondiagnosis of CTPA scans in this population.

The study was approved by our local Health Research Ethics Board (HREB) privacy safeguards were adhered to.

## 3. Results

There were 83 pregnant or postpartum patients included in this study. There were 31 (37%) pregnant patients, and 52 (63%) postpartum patients. The duration of pregnancy ranged from one week to 40 weeks pregnant, with an average duration of 24 weeks and two days. The majority of pregnant patients were in the second and third trimesters. There were six patients in the third trimester, 12 patients in the second trimester, and 13 patients in the third trimester. The postpartum patients ranged from zero to 42 days postpartum, with an average of nine days postpartum.

The pregnant and postpartum population had more nondiagnostic CTPAs than a comparable nonpregnant/postpartum population. Using our “objective” definition, 36 (43%) pregnant or postpartum patients achieved a nondiagnostic CTPA while 24 (26.9%) nonpregnant or postpartum patient CTPAs were nondiagnostic which is a significant difference (*p* < 0.05). The rate of nondiagnosis was also compared to the description of the study quality by the reporting radiologist. If the quality of the study was described as limited by the radiologist, this was taken as a nondiagnostic scan by “subjective” criteria. By this criterion, there were 27 (33%) nondiagnostic scans in the pregnant or postpartum group, while there were only 9 (10%) in the nonpregnant group which is also significantly different (*p* < 0.01).

The objective determination of nondiagnosis was established using two criteria: level of motion artefact and attenuation of the pulmonary artery, as described in the methods section. In the pregnant/postpartum population, motion artefact caused nondiagnosis in only five out of the 36 (14%) nondiagnostic scans, while attenuation of the pulmonary artery was responsible for nondiagnosis for all 36 (100%) of the nondiagnostic scans. In the nonpregnant/postpartum population, motion artefact was the reason for nondiagnosis in four out of 24 (16%) nondiagnostic scans. Level of pulmonary artery attenuation was the cause of nondiagnostic scan quality in 22 out of 24 (92%) scans.

The objective nondiagnostic rate was greater than the subjective rate, as described in Table 1. There were more objectively determined nondiagnostic CTPAs than subjective in the pregnant or postpartum population, although the results were comparable with 36 (43.4%) objective nondiagnostic CTPAs and 27 (32.5%) subjective nondiagnostic CTPAs in this population. The radiologist's opinion correlated with an objective nondiagnosis for 24 patients (66.7%). There were 12 (33.3%) CTPA studies that received nondiagnostic status by to our objective definition which were not identified as nondiagnostic by the reporting radiologist. On three occasions, the reporting radiologist determined a CTPA to be nondiagnostic that did not fit the objective criteria (3.6%); however, on all of these occasions, the motion artefact was given a grade of 2, indicating that segmental pulmonary arteries were obscured.

The objective determination of nondiagnostic scans in nonpregnant and non-post-partum patients compared to subjective determination is illustrated in Table 1. There were more objectively determined nondiagnostic scans (27.0%) than subjective (10.1%). The subjective determination of nondiagnosis correlated with objective determination 29.2% of the time. There were 17 (70.8%) objectively nondiagnostic scans that were not considered nondiagnostic by the radiologist's opinion. There were only two scans that were identified as nondiagnostic by the radiologist's opinion, which were not supported by our objective definition.

In the pregnant/postpartum population only 55 (66.27%) patients underwent a chest X-ray before their CTPA. There were 38 patients (69.10%) who had a normal chest X-ray and 17 (30.90%) who had an abnormal chest X-ray. The nondiagnostic CTPA rate was similar between patients who had abnormal and normal chest X-rays when using our objective criteria, as shown in Table 2. There were 16 (42.1%) patients with a normal chest X-ray who achieved a nondiagnostic CTPA. There were 8 (47.10%) patients with an abnormal chest X-ray who achieved a nondiagnostic CTPA. There was no significant difference between these results (*p*=0.73).

The results of CTPA nondiagnosis in nonpregnant/postpartum patients is shown in Table 2. In this population, 66 (74.1%) of patients underwent a chest X-ray prior to CTPA. There were 41 (62.1%) normal X-rays and 25 (37.8%) abnormal X-rays. According to our objective criteria, there were 10 (24.39%) patients with a normal chest X-ray who achieved a non diagnostic CTPA. There were 11 (44.0%) patients with an abnormal chest X-ray who achieved a nondiagnostic CTPA. The difference in nondiagnostic rate between normal and abnormal chest X-ray was larger, however still not significant (*p*=0.10).

The significance of using the objective or subjective criteria depends on which population you are evaluating. In the pregnant or postpartum population there is not significant difference in the nondiagnostic rates (objective 43%, subjective 33%, *p*=0.15), while in the nonpregnant or postpartum population, the difference is significant (objective 27%, subjective 10%, *p* < 0.01).

## 4. Discussion

The nondiagnostic rate of CTPA was significantly higher in the pregnant/postpartum population than in the nonpregnant/postpartum population using both criteria. This is consistent with the fact that the physiological changes related to hemodynamic status that pregnant patients undergo make it more difficult to obtain appropriately timed contrast enhancement, while pregnant patients often report feeling short of breath during pregnancy which may make obtaining a breath hold scan more difficult. The postpartum period was defined as within 6 weeks of delivery because that is the timeframe within which the physiologic hypercoagulability of pregnancy has been shown to increase the risk of PE [11].

Interestingly, although we used two separate criteria to determine objective nondiagnosis of CT scans, the level of attenuation of the pulmonary artery was almost exclusively responsible for determining the nondiagnostic scans. This is encouraging as it is something that could be potentially addressed by altering contrast administration and improving the timing of image capture. This could potentially reduce the amount of nondiagnostic scans in this population.

The nondiagnostic rate in both pregnant and nonpregnant arms of this study was higher than expected and is higher than found elsewhere in the literature, for example, one retrospective case-control study determined that CT scans were nondiagnostic in 12% of pregnant or postpartum women, compared to 0% of nonpregnant/postpartum patients [12]. Another group of researchers with a similar goal performed a five-year-long retrospective cohort study comparing CT nondiagnosis to VQ nondiagnosis. They determined that CT scans were nondiagnostic 17% of the time; however, if a normal chest X-ray was first collected from these populations, CT scans were nondiagnostic 30% of the time [13]. Another nine-year-long retrospective study found that of 43 pregnant patients who underwent CT scans for suspected pulmonary embolism, 19% were indeterminate [14]. Finally, one study reported an even higher nondiagnostic rate of CT scans. They found that CT scans were nondiagnostic in 35.7% of 25 pregnant women, compared to only 2.1% of nonpregnant women (*p* < 0.001) [15]. All of these studies relied on the radiologists' report to determine which scans were nondiagnostic, and the variability in the results may be the result of this. Objective criteria allow us to standardize reporting and begin to define the significance of a nondiagnostic CTPA scan, which is as yet unknown.

The most clinically relevant finding is the high rate of CTPA nondiagnosis in patients with normal chest X-rays (42.1%) which is the patient population that is typically considered for VQ scanning. This seems warranted, as this value is much higher than the rate of nondiagnosis that has been quoted in the literature for SPECT VQ [12, 15], particularly given that the techniques are known to be similarly accurate in the diagnosis of PE. CTPA is likely still the modality of choice in patients with abnormal chest X-rays given its greater ability to identify alternative causes of patient symptoms.

It is notable that there is no significant difference between the rate of nondiagnosis in pregnant and postpartum patients when using the objective criteria than there is when using the radiologists' subjective opinion. This may suggest that pregnancy status might bias the reporting radiologist to more willingly categorize a scan as nondiagnostic, which is another indication that objective criteria is required.

## 5. Limitations

There were some limitations to this study. The data were collected from different sites across the Eastern Health Authority, and we know that different CT scanners were used across the seven-year period. There is no way to prove consistency between different scanners from different manufacturers at multiple different sites. The protocols used at different sites may also be slightly different however should be broadly applicable to modern machines. This may have affected the quality of the CT scans and contributed to the nondiagnosis of some of them.

In addition, while we can state the rate of nondiagnosis of CTPAs in this population, it is difficult to compare our results directly with the literature values for VQ scans. This is because there is a paucity of studies that have used an objective reproducible measure of nondiagnosis for VQ scans.

Finally, interpretation bias is possible as all the imaging studies will be reviewed by researchers taking part in the study. This issue is avoided by employing objective criteria and having two radiologists agree on the categorization.

## 6. Conclusion

This is the first study to attempt to identify an objective method of determining CTPA nondiagnosis in pregnant and postpartum patients suspected of a PE. It shows the importance of having a standardized method of determining nondiagnosis, as it is likely that many factors can influence the radiologist's interpretation of study quality, potentially including patient pregnancy status. Given that the nondiagnostic rate is much higher in the pregnant/postpartum patient group for CTPA in patients with normal chest X-ray than reported rates for modern VQ, SPECT VQ scan should be considered first line imaging when investigating for PE in this population. This would also serve to limit the radiation dose to the patient's proliferating breast tissue.

## Figures and Tables

**Table 1:** Identification of nondiagnostic (ND) CTPA scans.

	Number of subjectively ND CTPAs	Number of objectively ND CTPAs	Frequency that subjectively ND scans correlated with objectively ND	Number of objectively ND CTPAs not identified by subjective identification	Number of subjectively ND scans not identified by objective definition
Pregnant/postpartum patients	27/83	36/83	24/36	12/36	3/83
	32.50%	43.40%	66.70%	33.30%	3.60%

Nonpregnant/postpartum patients	9/89	24/89	7/24	17/24	2/89
	10.1%	27.0%	29.2%	70.8%	2.2%

**Table 2:** The effect of normal chest X-rays on CTPA nondiagnosis (ND).

Pregnant/postpartum patients	Frequency of CXR completion prior to CT	Number of normal CXR	Number of ND CTPAs in patients with normal CXR
	55/83	38/55	16/38
	66.27%	69.10%	42.10%
		# abnormal CXR	# ND CTPAs in patients with abnormal CXR
		17/55	8/17.0
		30.90%	47.10%

Non pregnant/postpartum patients	Frequency of CXR completion prior to CT	Number of normal CXR	Number of ND CTPAs in patients with normal CXR
	66/89	41/66	10/41
	74.1%	62.1%	24.39%
		# abnormal CXR	# ND CTPAs in patients with abnormal CXR
		25/66	11/25
		37.80%	44.0%

## Data Availability

The patient information data used to support the findings of this study are restricted by the Health Ethics Review Board at Memorial University of Newfoundland in order to protect Patient Privacy. Data are available from Sarah Hogan, seh663@mun.ca, for researchers who meet the criteria for access to confidential data.
